# Silencing Rac1 and Prex1 Inhibit Epithelial–Mesenchymal Transition in Human Gastric Cancer Cells Induced by Transforming Growth Factor-β1

**DOI:** 10.5152/tjg.2023.23108

**Published:** 2023-09-01

**Authors:** Xinyan Gao, Xiaoyan Lin, Mengxin Lin, Yanqin Lan, Yao Wang, Riping Wu, Junde Li, Chuanyong Huang, Dongta Zhong

**Affiliations:** 1 Department of Medical Oncology, Fujian Medical University Union Hospital, Fuzhou, Fujian Province, China; 2 Department of Medical Oncology, Zhangzhou Municipal Hospital, Zhangzhou, Fujian Province, China

**Keywords:** Human gastric cancer cells, epithelial–mesenchymal transition, Rac1, Prex1, transforming growth factor β1

## Abstract

**Background/Aims:**

Transforming growth factor-beta can influence tumor cells, causing epithelial–mesenchymal transition and enhancing their invasion and metastasis ability. Rac1 protein could be used as an independent tumor diagnostic marker and survival predictor. Prex1 is closely related to cell metastasis. In this study, the impact of silencing Rac1 and Prex1 on transforming growth factor-beta 1-induced epithelial–mesenchymal transition and apoptosis of human gastric cancer cells MGC-803 and MKN45 was investigated.

**Materials and Methods:**

MGC-803 and MKN45 cells received recombinant transforming growth factor-beta 1 (rTGF-β1) treatments at various concentrations. Cell Counting Kit-8 kit was used to determine cell viability. Rac1 and Prex1 interference vectors were transfected into the rTGF-β1-treated MGC-803 and MKN45 cells. Cell apoptosis and migration were detected by flow cytometry and scratch test, respectively. Western blot was used to detect the epithelial–mesenchymal transition-related markers E-cadherin, N-cadherin, vimentin, and PDLIM2 expression levels.

**Results:**

The rTGF-β1 (10 ng/mL) could promote MGC-803 and MKN45 cell viability. Silencing Rac1 and Prex1 could increase E-cadherin and PDLIM2 expression, decrease N-cadherin and vimentin expression, inhibit cell viability and migration, and promote apoptosis in rTGF-β1-treated MGC-803 and MKN45 cells.

**Conclusion:**

Silencing Rac1 and Prex1 could inhibit epithelial–mesenchymal transition, reduce cell viability and migration, and promote apoptosis in human gastric cancer cells.

**Main Points**

- Silencing Rac1 and Prex1 could inhibit epithelial–mesenchymal transition (EMT), increase PDLIM2 expression, decrease cell viability and migration, and enhance apoptosis in human gastric cancer cells.
- Rac1 and Prex1 play a key role in cancer cells.
- This study provided new ideas for the treatment of EMT-related diseases.

## Introduction

Gastric cancer is one of the most common malignancy worldwide. The gastric cancer mortality rate is currently the second highest in the world.^1^ Invasion, metastasis, and postoperative recurrence account for the majority of gastric cancer patients’ deaths.^2,3^ Therefore, it is essential to study the mechanism of gastric cancer invasion and metastasis to improve the effectiveness of clinical treatment and the survival rate.^4^

Epithelial–mesenchymal transition (EMT) remarkably influences gastric cancer invasiveness in the tumor microenvironment, and drug resistance is easy to develop.^5,6^ During EMT, the epithelial cells lose polarity, adhesion with the surrounding cells decreases, and the movement and migration ability increases. The EMT has a significant function in embryonic development, damage repair, tissue homing and tumor invasion and metastasis.^7-9^

Zhao et al^10^ transfected Rac1 shRNA into Lovo colorectal cancer cells, and it showed that *Rac1 *gene downregulation inhibited lamellipodia formation and tumor cell migration in vitro. Rac1 protein could be used as an independent tumor diagnostic marker and survival predictor. Prex1 is a Rac1 exchange factor that binds and activates Rac1 by exchanging bound guanosine diphosphate (GDP) with guanosine triphosphate (GTP). It has been shown to be crucial in the spreading of breast cancer and is closely associated with cell metastasis.^11^ Transforming growth factor-β (TGF-β) can affect tumor cells via autocrine and regulate extracellular matrix via paracrine, causing EMT in tumor cells and enhancing invasion and metastasis abilities. The TGF-β is one of the main inducing factors of EMT.^12^

At present, the roles and mechanisms of Rac1 and Prex1 in gastric cancer cell EMT are still unknown. Therefore, in this study, we developed a model of TGF-β1-induced EMT in gastric cancer cells. The RNA interference vectors of *Rac1 *and *Prex1 *genes were constructed to investigate the impact of silencing Rac1 and Prex1 expression on EMT, apoptosis, and cell viability of TGF-β1-treated gastric cancer cells.

## Materials and Methods

### Cell Lines and Main Reagents

The sources for the cell line and main reagents are presented in Supplementary Table 1.

### Cell Culture and Establishment of Epithelial–Mesenchymal Transition Models of MGC-803 and MKN45 Cells

The MGC-803 and MKN45 cells were cultured at 37°C, 5% CO_2_ in Dulbecco’s Modified Eagle Medium (DMEM) containing 10% fetal bovine serum (FBS) and 1% penicillin–streptomycin. The adherent cells were treated for 24 hours with rTGF-β1 at various concentrations (0, 5, 10, 20, and 50 ng/mL).

### Cell Counting Kit-8 Assay

The cells were harvested, digested, resuspended, counted, and seeded in the 96-well plates (7000 cells/well). The cells were cultured in a humidified incubator with 5% CO_2 _at 37°C for 24 hours. Each well was added with the Cell Counting Kit-8 (CCK-8) solution (10 μL). The plate was kept in an incubator for 2 hours. A multifunctional enzyme label analyzer microplate reader was used to read the absorbance at 450 nm. The cell viability was determined.

### Construction of Rac1 and Prex1 RNA Interference Vectors

*Rac1 *(accession number: NM_006908.5) and *Prex1 *genes (accession number: NM_020820.4) in GenBank were selected as the analysis sequences. Three siRNA interference vectors were constructed (Table 1). They were transfected into MGC-803 and MKN45 cells, and western blot was used to verify the transfection efficiency.

### Cell Transfection

The MGC-803 and MKN45 cells were randomly allocated into blank control (Control), SiRac1 NC, Rac1 interference (SiRac1), SiPrex1 NC, Prex1 interference (SiPrex1), Rac1 + Prex1 interference (SiRac1 + SiPrex1), and SiRac1 + SiPrex1 + rTGF-β1 groups. The cells were cultured in the 6-well plates (2 × 10^5^ cells/well). When the density reached 70%, the cells were transfected using Lipofectamine 3000 kit according to the manual instruction. The rTGF-β1 was added after 48 hours of transfection.

### Cell Migration Detection

When the density of cells reached more than 90%, the cells in each well were scratched and washed thrice with phosphate-buffered saline (PBS) after discarding the culture medium. Then, this was added with serum-free medium and continued culture for 24 hours. The migration rate of the cells was measured based on the scratch width corresponding to 0-24 hours. Cell migration = [(scratch width at 0 hour −scratch width at 24 hours)/scratch width at 0 hour] × 100%. The photos were opened with Image J. The scratch width at 0 and 24 hours at the same site in each photo were measured and calculated by the above formula. Each group had 5 replicates.

### Western Blot

Lysis buffer was added to the cells collected and centrifuged for 5 minutes at 4°C (10 000 rpm). The supernatant was collected for total protein. The bicinchoninic acid (BCA) was used to determine the protein concentration. Proteins (50 μg per lane) were loaded, separated with 12% sodium dodecyl sulfate–polyacrylamide gel electrophoresis, and electrotransferred to the polyvinylidene difluoride membrane. The membrane was rinsed with Tris-buffered saline (TBS) for 10-15 min and immersed in Tris-buffered saline with Tween 20 (TBS/T) blocking buffer containing 5% (w/v) skimmed milk powder. Primary antibodies for E-cadherin, N-cadherin, PDLIM2, and vimentin were added and incubated at 4°C overnight. Then, the membrane was rinsed thrice with TBS/T, horseradish peroxidase-labeled secondary antibody was added and incubated at room temperature for 1 hour. Enhanced chemiluminescence was used to determine the protein bands. Quantification was conducted using ImageJ software as a ratio to glyceraldehyde-3-phosphate dehydrogenase (GAPDH).

### Flow Cytometry for Cell Apoptosis

The apoptosis was analyzed using Annexin V–fluorescein isothiocyanate (FITC) Analysis Kit based on the manufacturer’s instructions. The cells were centrifuged and resuspended in 300 μL of 1× binding buffer. A total of 1 × 10^6^/mL cells were collected. Annexin V-FITC (3 μL) and propidium iodide–phycoerythrin (5 μL) were added and incubated in the dark at 4°C for 10 min. Then, the cells were added with 200 μL of precooled 1× binding buffer. Flow cytometry was used to determine the apoptosis rate. The experiment was repeated thrice.

### Statistical Analysis

The data were analyzed using GraphPad Prism 7, and presented as mean ± standard deviation (SD). The differences among groups were evaluated by one-way and two-way analysis of variance. *P* < .05 was considered significant.

## Results

### Screening for Optimal rTGF-β1 Concentration

**Figure 1.**
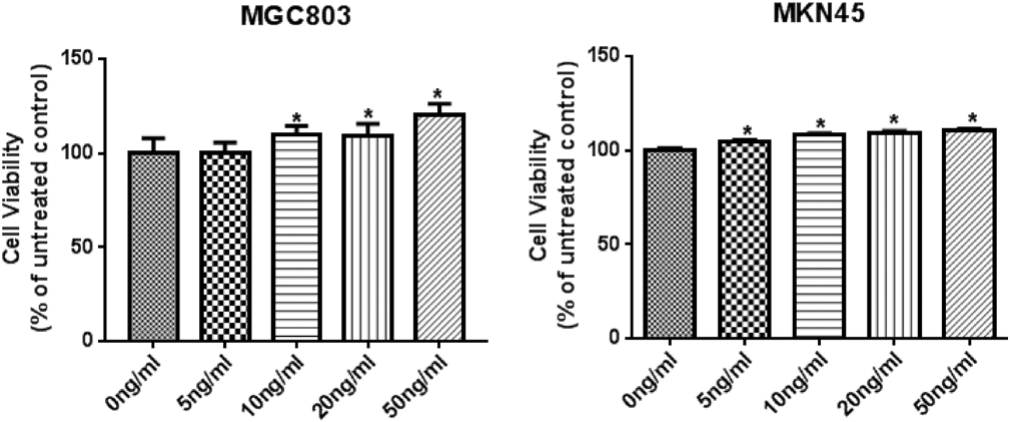
Screening for optimal rTGF-β1 concentration. ^*^ *P* < .05 vs. Control.

The MGC-803 and MKN45 cells were subjected to rTGF-β1 treatment for 24 hours. The cell viability was determined with the CCK-8 kit. Compared with the control group, 10, 20, and 50 ng/mL rTGF-β1 remarkably enhanced MGC-803 and MKN45 cell viability (Figure 1). At 10 ng/mL rTGF-β1, the cell viability was significantly increased in both MGC-803 and MKN45 cells compared to control, and subsequent increase in concentration had little effect on the cell viability; therefore, 10 ng/mL rTGF-β1 were selected for subsequent experiments.

### Transfection Efficiency of Rac1 and Prex1 Small Interfering RNAs (siRNAs)

**Figure 2.**
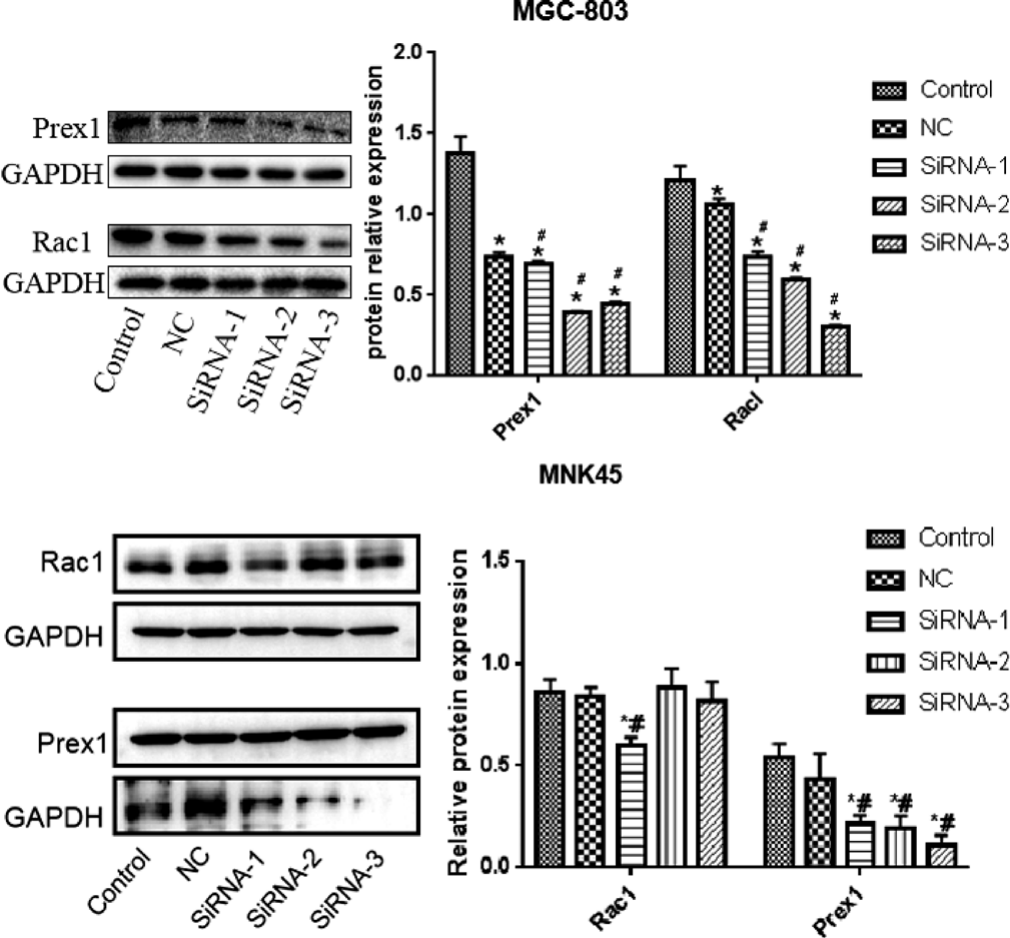
Rac1 and Prex1 expression levels detected by western blot. ^*^ *P* < .05 vs. control; ^#^ *P* < .05 vs. NC.

The transfection efficiencies of Rac1 and Prex1 siRNAs in MGC-803 and MKN45 cells were determined by their expression levels detected by western blot. Compared with the control and NC groups, the Rac1 and Prex1 protein expression levels in the interference group in MGC-803 cells were remarkably decreased, and the interference effects of SiPrex1-3 and SiRac1-3 were the best, while in MKN45 cells, SiRac1-1 and SiPrex1-3 showed the best interference effect (Figure 2).

### Effects of Rac1 and Prex1 on MGC-803 and MKN45 Cell Viability

**Figure 3.**
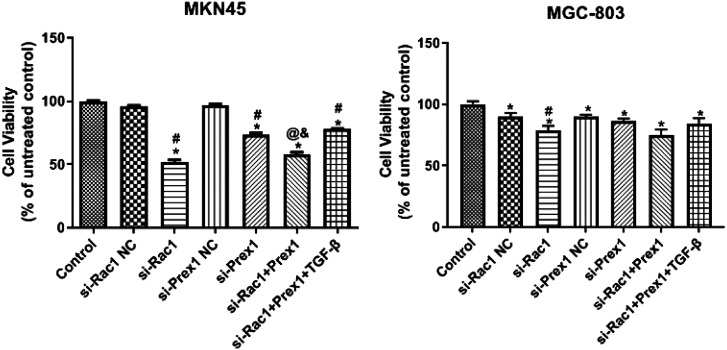
Effects of Rac1 and Prex1 on the viability of MGC-803 and MKN45 cells. ^*^ *P* < .05 vs. control; ^#^ *P* < .05 vs. NC; ^@^ *P* < .05 vs. si-Rac1; ^&^ *P* < .05 vs. si-Prex1.

Compared with the control group, all treatment groups inhibited MGC-803 and MKN45 cell viability, and the inhibition degrees of the SiRac1 group and SiRac1+ SiPrex1 group were higher than those of the other treatment groups (Figure 3).

### Effects of Rac1 and Prex1 on Migration of MGC-803 and MKN45 Cells

**Figure 4.**
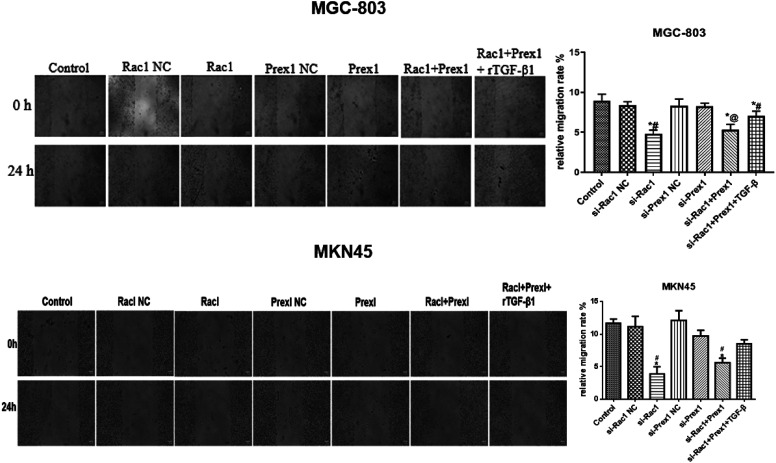
Effects of rTGF-β1, Rac1, and Prex1 on the migration of MGC-803 and MKN45 cells. ^*^ *P* < .05 vs. control; ^#^ *P* < .05 vs. NC; ^@^ *P* < .05 vs. si-Rac1.

Compared with the control group, the migration of MGC-803 and MKN45 cells was inhibited in the SiRac1 group, SiPrex1 group, SiRac1 + SiPrex1 group, and Rac1 + Prex1 + rTGF-β1 group. The Rac1 interference group and Rac1 + Prex1 interference group demonstrated higher inhibition degrees than those of the other treatment groups (Figure 4).

### Effects of Rac1 and Prex1 on Apoptosis in MGC-803 and MKN45 Cells

**Figure 5.**
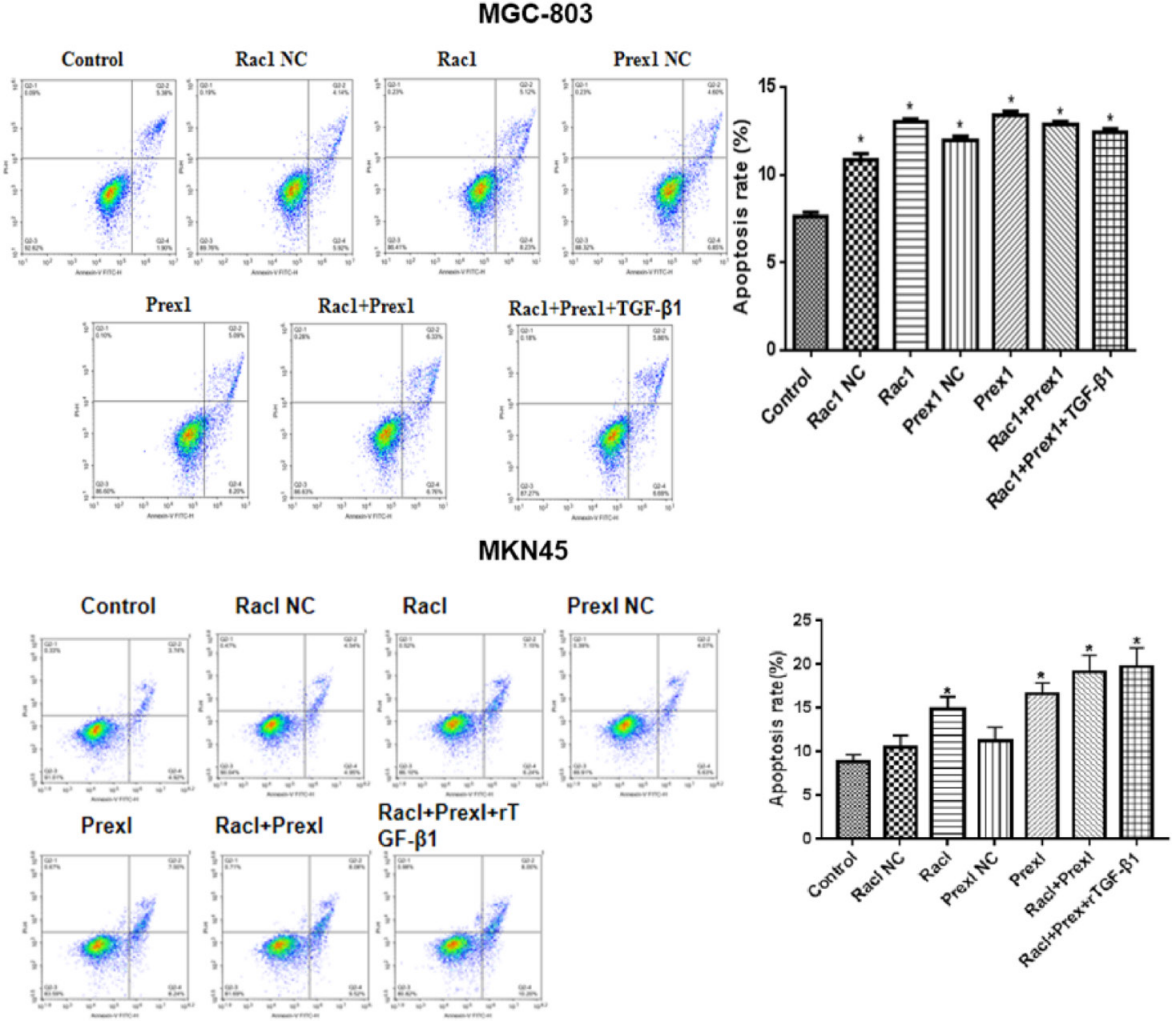
Effects of rTGF-β1, Rac1, and Prex1 on the apoptosis of MGC-803 and MKN45 cells. ^*^ *P* < .05 vs. Control.

Compared with the control group, the SiRac1, SiPrex1, SiRac1 + SiPrex1 interference, and Rac1 + Prex1 + rTGF-β1 groups promoted MGC-803 and MKN45 cell apoptosis (Figure 5).

### Effects of Rac1 and Prex1 on the Epithelial–Mesenchymal Transition-Related Markers Expression in MGC-803 and MKN45 Cells

**Figure 6.**
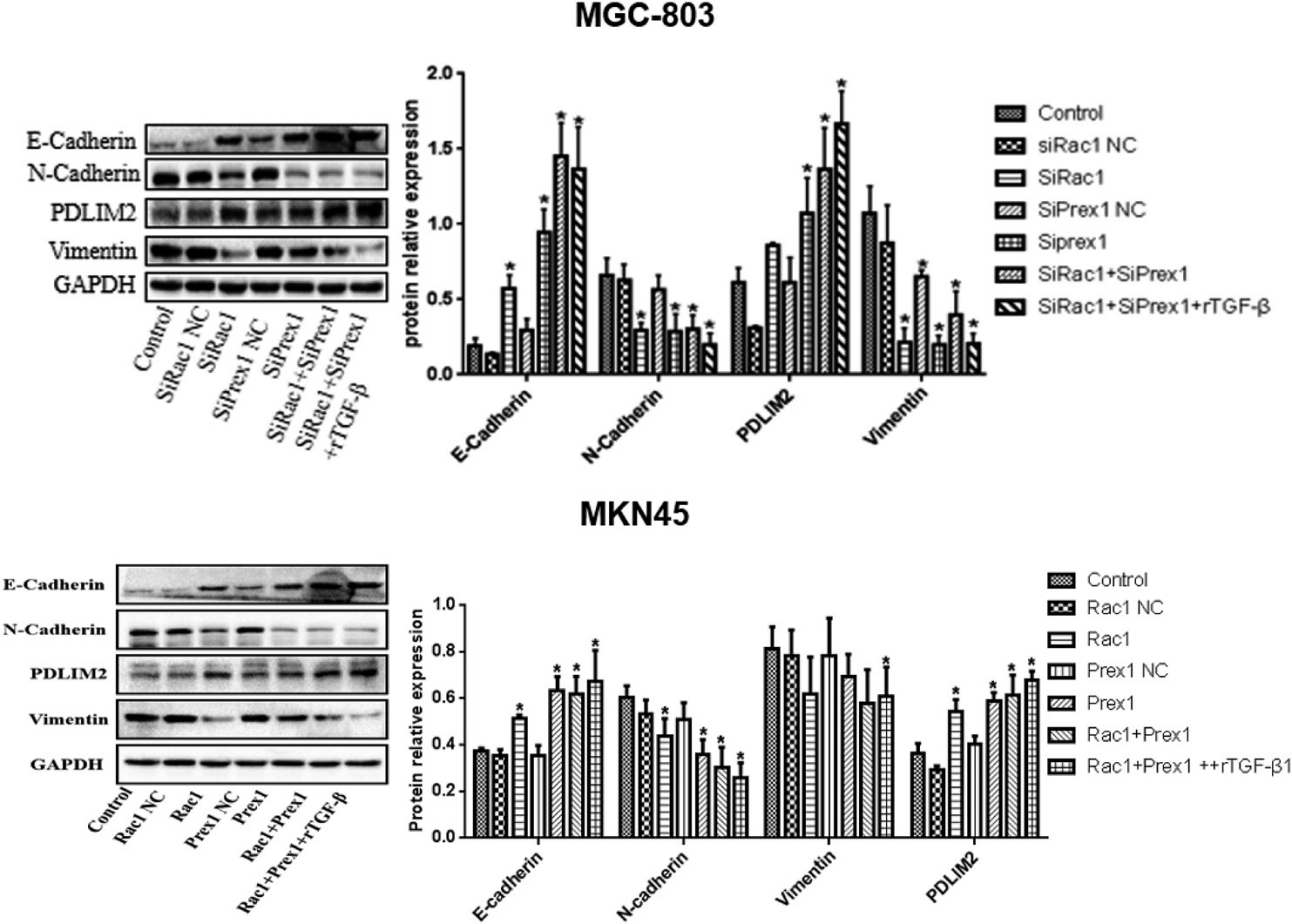
Expression of E-cadherin, N-cadherin, vimentin, and PDLIM2 determined by western blot. ^*^ *P* < .05 vs. control.

Compared with the control group, the E-cadherin and PDLIM2 expression was remarkably increased, while N-cadherin and vimentin were remarkably decreased in the SiRac1, SiPrex1, SiRac1+SiPrex1 interference, and Rac1 + Prex1 + rTGF-β1 groups in MGC-803 and MKN45 cells (Figure 6).

## Discussion

Invasion and metastasis are the primary barriers to treating malignant tumors and the leading cause of death.^13^ However, the mechanism of cancer invasion and metastasis is still unclear. Gastric cancer has become one of the most important diseases threatening human health. The Rac1 and Prex1 were found to be positively correlated with tumor cell invasion and metastasis.^11,14-16^ Therefore, we explored the effect of Rac1 and Prex1 on the invasion and metastasis of gastric cancer cells.

The Rac1 influences the invasion and metastasis of tumor cells by regulating the cytoskeleton reorganization and participates in modulating the cell cycle and apoptosis.^14^ The Prex1 is a Rac-guanine nucleotide exchange factor (GEF). In 2016, Liu et al^16^ found that the activity of PREX1-Rac-GEF is crucial for the growth of cells dependent on Prex1 and the growth of transplanted tumors, and it may become a therapeutic target for breast cancer. Malliri et al^17^ reported that persistent activation of Q61L Rac1 could cause the malignant transformation of mouse fibroblasts. The introduction of active Rac1b mutant into dormant Swiss 3T3 cells could accelerate the cell process in the G1–S phase and increase DNA synthesis, whereas inhibitory Rac1b mutant could inhibit the process of the cell cycle. In this study, we found that silencing *Rac1 *and *Prex1 *genes by RNA interference considerably inhibited MGC-803 and MKN45 cell migration and promoted apoptosis.

The EMT is a crucial process in the lung, liver, gastric, and colon cancer early stage of invasion and metastasis.^18^ The occurrence of EMT is affected by many factors, among which TGF-β1 is the key factor. The TGF-β1 can induce EMT in some epithelial cells through Smad or non-Smad signaling pathway.^19-21^ In the present study, high rTGF-β1 concentration promoted MGC-803 cell viability, but the MGC-803 cell viability was not affected by low rTGF-β1 concentration, indicating that the expression of EMT transcription factors may need to accumulate to a certain concentration to bind with the promoter of E-cadherin to inhibit its expression, resulting in loose cell junctions.^22^ During EMT, the molecular markers changed, E-cadherin was transformed into N-cadherin, and the expression of vimentin was increased, which promoted cell mobility, invasion, and metastasis. N-cadherin is negatively correlated with E-cadherin.^23-25^ The TGF-β1 can increase p-LIMK and p-cofilin expression, increase F-actin polymerization, downregulate the E-cadherin level, and upregulate the vimentin level.^26^ In the present study, we found that Rac1 and Prex1 siRNAs remarkably decreased the levels of N-cadherin and vimentin expression and increased E-cadherin expression. In addition, EMT was inhibited in rTGF-β1-induced gastric cancer. These results suggested that Rac1 and Prex1 may act as TGF-β1 downstream target genes and participate in the EMT pathway of MGC-803 and MKN45 cells. From the effects of interfering with Rac1 and Prex1 alone, it can be seen that the effect of interference with Rac1 on cell viability, apoptosis, and migration of gastric cancer cells is significantly higher than that of the Prex1 interference group, indicating that Prex1 interference has little effect on Rac1 in gastric cancer cells. On the other hand, the sensitivity to Rac1 and Prex1 interference in MGC-803 cells and MKN45 cells is also inconsistent. The Rac1 and Prex1 interference has more significant effects on the cell viability, migration, and apoptosis in MKN45 cells, indicating that the sensitive factors of EMT in cells from different sources may also vary.

Drug resistance is a characteristic of cancer cells.^27^ The PDLIM2 protein contains PDZLIM domain, and its main function is to promote the NF-κB RelA and STAT3 ubiquitination and proteasome degradation.^28,29^ By inhibiting NF-κB/RelA and STAT3, PDLIM2 increased the gene expressions that participated in antigen presentation and T cell activation and inhibited multidrug resistance genes and cancer-related genes, making cancer cells vulnerable to immune attack and treatment.^30^ Silencing *Rac1 *and *Prex1 *genes remarkably increased PDLIM2 expression in MGC-803 and MKN45 cells, indicating that *Rac1 *and *Prex1 *gene interference can repair cancer cells.

One of the limitations of this study is that it mainly explored the effect of silencing Rac1 and Prex1 on EMT in human gastric cancer in vitro. Further studies may investigate the mechanisms* in vivo*. Another limitation is that SiRac1 + rTGF-β1 and SiPrex1 + rTGF-β1 groups were not established in this study. Forming SiRac1 + rTGF-β1 and SiPrex1 + rTGF-β1 groups alone to examine the effect of silencing each protein in the presence of recombinant TGF-β1 may enrich the content of the study and the impact of Rac1 and Prex1 can be more directly seen. However, since Prex1 is the exchange factor of Rac1, and based on the existing data of the article that the effect of Rac1 interference on gastric cancer cells is higher than that of Prex1, and the interference effect of the two genes is also obvious, while rTGF-β1 treatment can counteract the effects of both interference, this can also explain that the presence of recombinant TGF-β1 can activate EMT, and Rac1 and Prex1 are also closely related to EMT.

In conclusion, interference with Rac1 and Prex1 could inhibit MGC-803 and MKN45 cells EMT, promote apoptosis, and upregulate PDLIM2 expression. The Rac1 and Prex1 play crucial roles in cancer cells. This study provided new ideas and approaches for the treatment of EMT-related diseases.

## Figures and Tables

**Table 1:** siRNAs used in the Study

siRNA name	siRNA sequences
Rac1 (human) siRNA-1	CCGAAUGAAGCGUUGCCAUTTAUGGCAACGCUUCAUUCGGTT
Rac1 (human) siRNA-2	UCUGUCAAAUGCAGUAGAUTTAUCUACUGCAUUUGACAGATT
Rac1 (human) siRNA-3	GGUUGGUAUUAUCAGGAAATTUUUCCUGAUAAUACCAACCTT
Rac NC	UUCUCCGAACGUGUCACGUTTACGUGACACGUUCGGAGAATT
Prex1 (human) siRNA-1	CCACAACACGGCCAAGAAUTTAUUCUUGGCCGUGUUGUGGTT
Prex1 (human) siRNA-2	CCAUCAAUGCUCUCCUCAATTUUGAGGAGAGCAUUGAUGGTT
Prex1 (human) siRNA-3	GCAUGAAAUCUCUGGUUUATTUAAACCAGAGAUUUCAUGCTT
Prex1 NC	UUCUCCGAACGUGUCACGUTTACGUGACACGUUCGGAGAATT

**Supplementary 1:** Sources for Cell Lines and Main Reagents

Cell Lines and Main Reagents	Sources
MGC-803 (catalog no.: TCHu 84)	Chinese Academy of Sciences
MKN45 (CL-0292)	Procell Life Science & Technology Co., Ltd.
OPTI-MEM® (31985-062)	Gibco
Complete DMEM (KGM12800S-500)	Nanjing KeyGen Biotech Co., Ltd.
Trypsin EDTA digestive solution (T1300)	Solarbio
Lipofectamine 3000 (L3000015)	Invitrogen™
rTGF-β1 (10804-H08H1)	Sino Biological
CCK-8 kit (KGA317)	Nanjing KeyGen Biotech Co., Ltd.
BCA Protein Assay Kit (CW0014S)	Beijing ComWin Biotech Co., Ltd.
Enhanced chemiluminescence	Perkin-Elmer Inc.
1% penicillin–streptomycin	Hyclone Laboratories
AnnexinV–FITC Analysis Kit	Beyotime Biotechnology
Multimode microplate reader (SuPerMax 3100)	Shanghai Flash Spectrum Biotechnology Co., Ltd.
